# Biological Properties of Promising Poly(isobutyl cyanoacrylate) Nanoparticles Coated with Fucoidan, Levan, or Chitosan

**DOI:** 10.1021/acsomega.6c08464

**Published:** 2026-10-09

**Authors:** Iago Dillion Lima Cavalcanti, Andreza Tallyne de Aguiar Silva, Leandro Paes de Brito, Clarice Beatriz Gonçalves Silva, Fabrício Oliveira Souto, Romero Marcos Pedrosa Brandão Costa, Otília Deusdênia Loiola Pessoa, Eloisa Berbel Manaia, Gilles Ponchel, Nereide Stela Santos Magalhães, Mariane Cajubá de Britto Lira Nogueira

**Affiliations:** † Instituto Keizo-Asami (iLIKA), Universidade Federal de Pernambuco (UFPE), Recife 50670-901, Brazil; ‡ Laboratório de Nanotecnologia, Biotecnologia e Cultura de Células, Centro Acadêmico de Vitória, Universidade Federal de Pernambuco (CAV/UFPE), Recife 55608-680, Brazil; § Laboratório de Avanços em Biotecnologia de Proteínas e Tecnologia de Bioativos, Universidade Federal Rural de Pernambuco (UFRPE), Recife 52171-900, Brazil; ∥ Laboratório Integrado de Biotecnología Aplicada à Saúde, Universidade de Pernambuco (UPE), Recife 52171-900, Brazil; ⊥ Laboratório de Análise Fitoquímica de Plantas Medicinais II, Departamento de Química Orgânica e Inorgânica, Universidade Federal do Ceará, Fortaleza 60020-181, Brazil; # Faculté de Pharmacie Institut Galien Paris-Saclay - UMR-CNRS 8612, Université Paris-Saclay, Orsay 91400, France

## Abstract

The objective of this study was to comparatively evaluate the biological behavior of poly­(isobutyl cyanoacrylate) nanoparticles coated with fucoidan, chitosan, or levan. We evaluated their *in vitro* hemocompatibility, coagulation profile, platelet aggregation, cytotoxicity, in vitro wound-healing activity, and stability during simulated gastrointestinal digestion. The three formulations exhibited distinct biological responses depending on the polysaccharide used. All nanosystems showed nonhemolytic action under the tested conditions. In coagulation and platelet assays, Chi-NPs displayed procoagulant tendencies and Lev-NPs also promoted coagulation, whereas Fuc-NPs demonstrated concentration-dependent anticoagulant effects. Cytotoxicity results varied across the assays, with the MTT assay showing cytotoxicity and apparent preservation of cell morphology, whereas the nanoparticles were not found to be cytotoxic using the flow cytometry technique. All formulations supported fibroblast migration in the wound-healing model, with Fuc-NPs showing the most pronounced effect. During simulated digestion, only Chi-NPs exhibited marked instability, with substantial size variation (123.2 ± 2.18 nm to 653.9 ± 534.41 nm) upon exposure to digestive enzymes. These findings highlight the importance of *in vitro* characterization as an initial step toward understanding how formulation-specific physicochemical properties influence biological behavior. However, the results are not sufficient to establish safety or suitability for *in vivo* administration. The differences observed among the formulations reflect not only the chemical identity of the polysaccharides but also the intrinsic physicochemical characteristics of each nanoparticle. Thus, this study should be interpreted as a comparative characterization of three distinct PIBCA systems.

## Graphical abstract

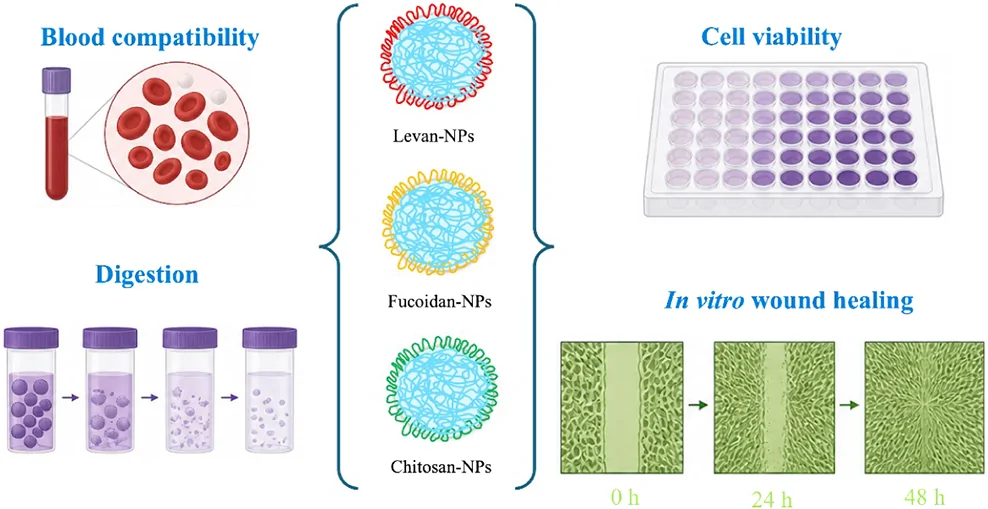

## 1 Introduction

Polysaccharides have been widely used in the development of nanocarriers due to their versatility, solubility, low toxicity, and pharmacological properties, which make them good polymers for the development of nanosystems with great potential for obtaining a new pharmaceutical product.^1,2^ Several studies have demonstrated the benefits of using polysaccharides in the composition of nanocarriers.^3,4^ This reflects the growing interest in polysaccharide-based nanostructures and their relevance to nanomedicine.

The benefits of nanosystems for the treatment of diseases is remarkable, as is the extent to which pharmaceutical nanotechnology has revolutionized medicine in the development of safe and effective drugs, which have been highlighted in recent years by the COVID-19 pandemic,^3,4^ as well as the inclusion of nanoencapsulated chemotherapy drugs for the treatment of cancer such as Doxil and Ambraxane.^5,6^ Over the past decade, extensive research has advanced our understanding of nano–bio interactions, including protein corona formation, biodistribution, immune responses, pharmacokinetics, and nanomaterial toxicity. However, despite this progress, important knowledge gaps remain for specific classes of nanocarriers, particularly polymeric systems whose surface chemistry can be extensively modified.^7,8^

Poly­(isobutyl cyanoacrylate) (PIBCA) nanoparticles represent one such system. Although PIBCA nanoparticles have been investigated since the 1980s, most studies have focused on how surface chemistry influences interactions with biological components—such as cell membrane binding, endocytic pathways, plasma protein adsorption, and complement activation.^9–15^ Far fewer studies have examined how PIBCA nanoparticles behave when coated with structurally distinct polysaccharides or how these coatings modulate their biological compatibility, stability, and functional properties. This is particularly relevant because polysaccharides differ markedly in molecular architecture, charge distribution, and biological activity, which may influence nanoparticle behavior in complex biological environments.

Our research group has worked with PIBCA nanoparticles for more than 15 years, exploring how different polysaccharides can be incorporated onto their surface to modulate their physicochemical and biological characteristics. In this manuscript, we investigate three PIBCA formulations coated with fucoidan, levan, or chitosan—polysaccharides with distinct chemical compositions and known bioactivities. These coatings offer opportunities to tailor nanoparticle–biological interactions and potentially expand the applicability of PIBCA-based systems.

It is important to note that although the nanoparticles were prepared using the same method, the resulting formulations differ not only in the identity of the polysaccharide but also in parameters such as size, surface charge, and potential coating density. These factors can significantly influence biological interactions. Therefore, this study was designed as a comparative characterization of three distinct PIBCA formulations.

## 2 Materials and Methods

### 2.1 Materials

Fucoidan (from *Fucus vesiculosus*, Mw 44,700 Da), dimethyl sulfoxide (DMSO), pancreatic trypsin, 3-(4,5-dimethyl-2-thiazolyl)-2,5-diphenyl-2H-tetrazolium bromide (MTT), glutamine, fetal bovine serum (FBS), and RPMI 1640 medium were purchased from Sigma-Aldrich (USA). The cell lines (macrophages J774.A1, VERO, and fibroblast L929) were obtained from the Cell Bank of Rio de Janeiro (BCRJ) (Brazil). Water-soluble chitosan (Mw 20,000 g/mol) was obtained from Amicogen (Jinju, South Gyeongsang, South Korea), isobutylcyanoacrylate (IBCA) from Afinitica (Barcelona, Spain), and the pT, aPTT, and CaCl_2_ coagulation kits from BIOS Diagnóstica (São Paulo, Brazil). The enzyme lipase was purchased from Galena (São Paulo, Brazil), amylase from Irial Mag (São Paulo, Brazil), and pepsin from SM Empreendimentos Farmacêuticos (São Paulo, Brazil). Levan (Mw 275.95 kDa)^16^ was extracted by the group from the bacterium ZAG 12 *Zymomonas mobilis* present in the bacterial bank of the Department of Antibiotics at the Federal University of Pernambuco (UFPE), following the methodology described by Silva et al.,^10^ and oncocalyxone A (oncoA) was supplied by Professor Dr. Otília Pessoa of the Federal University of Ceará.

### 2.2 Obtaining Nanoparticles and Characterizing Them

Lev-NPs, Chi-NPs, and Fuc-NPs were obtained using the anionic emulsion polymerization (AEP) technique, following the methodology described by Lira et al.^11^ and Cavalcanti et al.^12^ Briefly, 50 mg of the polysaccharide (levan, fucoidan, or chitosan) was dissolved in 5 mL of ultrapure water and the pH corrected to 2.5 with 1 M HCl. After vigorous stirring (1500 rpm), 50 μL of IBCA was added to start the polymerization and formation of the nanoparticles. For the fluorescence studies, oncoA (1 mg/mL), a fluorescent marker, was encapsulated. The oncoA solution was added 5 min after the addition of IBCA, as recommended by Cavalcanti et al.^12^ After 1 h of reaction, the nanoparticles were purified by dialysis using a Spectra-Por dialysis membrane with a molecular weight cutoff of 100,000 g/mol (MWCO) (Biovalley, Marne-la-Vallée, France) in 1 liter of ultrapure water, with two water changes during the first hour and dialysis continued overnight with stirring at 300 rpm. The Zetasizer nano ZS90 (Malvern Instruments, USA) was used to characterize the nanoparticles in terms of size, polydispersity index (PDI), and surface charge. To do this, the nanoparticles were diluted in ultrapure water or 1 mM NaCl in a ratio of 1:20 and read at a temperature of 25 °C and an angle of 90 °. The analyses were performed in triplicate.

These nanocarriers were developed specifically for the analyses described in this study as new, independent batches and were characterized to confirm the reproducibility of the previous batches. The amount and surface density of the adsorbed polysaccharides were not quantified, a fact that should be considered when interpreting the biological results.

#### 2.2.1 Morphological Aspects of Nanoparticles

The morphologies of Lev-NPs, Chi-NPs, and Fuc-NPs were evaluated using a transmission electron microscope (TEM) (Tecnai G2 Spirit BioTWIN). To do this, the nanoparticles were diluted in ultrapure water in a ratio of 1:10 v/v and deposited on a 200-mesh copper grid. Before observation under a microscope, the grid was dried in a desiccator.

### 2.3 Blood Compatibility Studies

For the blood compatibility study, 4 mL of blood was collected from five healthy adult volunteers and inserted into a tube containing the anticoagulant sodium citrate. The blood was then diluted in 5 mL of phosphate-buffered saline (PBS). The blood containing the anticoagulant was used for hemolytic potential and coagulation studies. Blood without anticoagulant was used for coagulation kinetics and platelet aggregation studies.^17^ This study was approved by the Human Research Ethics Committee of UFPE, Brazil (CAAE 25179719.1.0000.5208).

#### 2.3.1 Hemolytic Toxicity Assay

The evaluation of the hemolytic potential was carried out according to the studies by Li et al.^18^ and Golafshan et al.,^19^ with some adaptations. Briefly, the dispersions containing Lev-NPs, Chi-NPs, and Fuc-NPs were diluted in PBS at concentrations of 400, 200, 100, 50, 25, and 12.5 μg/mL and incubated for 30 min at 37 °C. After incubation, whole blood diluted in PBS was added at a ratio of 1:100. PBS was used as a negative control and distilled water as a positive control. After adding the blood, the samples were returned to the oven for 1 h at 37 °C and then centrifuged for 5 min at 2500 rpm. Finally, the supernatant was removed, and the absorbance was read at 540 nm in an ELISA reader (Thermo Fisher Scientific, USA). The degree of hemolysis was expressed according to eq 1:

1 $$\mathrm{Hemolysis}\left(\%\right)=\frac{\left(\mathrm{Sample absorbance}-\mathrm{NC}\right)}{\mathrm{PC}-\mathrm{NC}}\times 100$$

NC= absorbance of the negative control; PC= absorbance of the positive control.

#### 2.3.2 Coagulation Studies

For the coagulation study, blood containing the anticoagulant (sodium citrate) was centrifuged at 1000 rpm for 10 min to obtain plasma. The plasma and nanoparticle dispersions diluted in PBS at concentrations of 400, 200, 100, 50, 25, and 12.5 μg/mL were then kept in the instrument (Coagulometer, 4-channel, Channels Semi-Automatic–MEDMAX, MaxClot) at a temperature of 37 °C. pT (TP CLOT, BIOS Diagnóstica, Brazil), aPTT (TTPA CLOT, BIOS Diagnóstica, Brazil), and CaCl_2_ reagents were used to obtain the coagulation time for the prothrombin time (pT) and activated partial thromboplastin time (aPTT) analyses.

##### 2.3.2.1 Coagulation Kinetics

The coagulation kinetics study was followed by dilution of the nanoparticles at concentrations of 400 and 12.5 μg/mL in PBS. The rapidly collected and diluted blood was distributed in the samples at a ratio of 1:100 and incubated for 1 h at 37 °C. At 1, 5, 15, 30, 60, 90, and 120 min, the samples were washed with distilled water to break up the free red blood cells and centrifuged at 2500 rpm for 5 min to separate the clot. The supernatant was removed, and its absorbance measured at 540 nm.^17^

##### 2.3.2.2 Evaluation of Clot Formation

To evaluate the process of clot formation, the nanoparticles were labeled with oncoA and incubated with platelet-rich plasma (ratio 1:1 v/v) in a 24-well plate containing coverslips (8 mm in diameter). At 5, 15, 60, and 120 min, the coverslips were removed and 400 μL of 4% paraformaldehyde was added for 20 min at 4 °C. After incubation, the coverslips were washed twice with PBS and 400 μL of ammonium chloride (50 mM) was added and incubated for 10 min. Finally, the samples were washed again with PBS and the coverslips fixed on slides and analyzed under a fluorescence microscope (Zeiss Axio, Imager.M2m, 20× objective, N.A. = 0.80), assessing fluorescence in the Alexa 488 (excitation length of 470/40 nm and emission of 525/50 nm), DAPI (excitation length of 350/50 nm and emission of 460/50 nm), and Rhodamine (excitation length of 550/25 nm and emission of 605/70 nm) filters.

#### 2.3.3 Platelet Aggregation

In the platelet aggregation study, diluted blood without anticoagulant was centrifuged at 1000 rpm for 10 min to obtain platelet-rich serum. The nanoparticle dispersion (Lev-NPs, Chi-NPs, or Fuc-NPs) and platelet-rich serum in a 1:1 ratio were added to a 24-well plate containing a round coverslip (8 mm in diameter) and incubated for 2 h at 37 °C. PBS was used as a control. The supernatant was then removed, and 400 μL of 4% paraformaldehyde was added for 1 h at 4 °C to fix the platelets. The samples were then dehydrated with 30, 50, 70, and 90% ethanol solutions. Finally, the laminae were fixed in stubs containing carbon, metallized with gold and palladium (DESK II, Denton Vacuum), and analyzed using a scanning electron microscope (SEM) (MIRA3, TESCAN).^17^

### 2.4 Cytotoxicity of Nanoparticles in Immortalized Cell Lines

The cytotoxicity study was carried out with 3 immortalized cell lines (macrophages J774.A1, VERO, and fibroblast L929). The immortalized strains were cultured in 75 cm^2^ bottles with RPMI 1640 medium supplemented with 10% FBS, 1% glutamine (200 mM), and 1% antibiotics (penicillin and streptomycin).^11^

The cell lines were plated in 96-well plates at a concentration of 1 × 10^4^ cells per well and incubated for 24 h at 37 °C and 5% CO_2_ for cell adhesion. To assess cytotoxicity, the nanoparticles were added at concentrations of 400, 200, 100, 50, 25, and 12.5 μg/mL diluted in supplemented RPMI 1640 medium. The plates were then incubated for 24 h, and after incubation, 20 μL of MTT solution (4 mg/mL) was added and incubated again for 2 h at 37 °C and 5% CO_2_. Finally, the supernatant was removed and 100 μL of dimethyl sulfoxide (DMSO) was added to dissolve the formazan crystals formed. The results were read on a microplate reader at a wavelength of 570 nm.

### 2.5 Cell Viability of Nanoparticles in PBMCs

A cytotoxicity study was carried out on peripheral blood mononuclear cells (PBMCs) isolated from five healthy human volunteers respecting the guidelines of the human ethics committee approved by the ethics committee of UFPE, Brazil (CAAE 25179719.1.0000.5208). To isolate the PBMCs, the collected blood was added to ACD (citric acid, sodium citrate, and dextrose) tubes. Then, 8 mL of the whole blood was added to a tube containing 7 mL of Ficoll-Paque to obtain the ring of PBMCs after centrifugation (30 min at 2100 rpm). The cells were washed twice with PBS + 2% FBS and then resuspended in 1 mL of RPMI 1640 medium supplemented with 10% FBS and 1% penicillin/streptomycin. The cells were counted using a Neubauer hemocytometer with trypan blue dye to obtain a concentration of 1 × 10^4^ cells/well in a 96-well plate.^20^

After plating, the nanoparticle dispersions were added at concentrations of 400, 200, 100, 50, 25, and 12.5 μg/mL and incubated for 24 h at 37 °C and 5% CO_2_. Then, 20 μL of MTT was added, and the cells were incubated again for 2 h (37 °C and 5% CO_2_). After incubation, the supernatant was removed and 100 μL of DMSO was added, followed by reading on a microplate reader at 570 nm.

For the flow cytometer studies, after exposure to the formulations for 24 h, the cells were washed with PBS, resuspended with the cell viability marker LIVE/DEAD, and incubated for 30 min at 37 °C in the dark. Finally, the cells were washed again with PBS and analyzed in a flow cytometer (BD FACSymphony A5).

### 2.6 *In Vitro* Wound Healing

For the *in vitro* healing assay, L929 fibroblast cells were cultured in a 24-well plate at a concentration of 1 × 10^6^ cells/well and incubated at 37 °C and 5% CO_2_ with RPMI 1640 medium supplemented with 10% FBS, 1% glutamine (200 mM), and 1% antibiotics (penicillin and streptomycin). After cell adhesion and confluence of 80–90% was observed, the cell monolayer was scraped using a tip (10–100 μL) to obtain a wound. The wells were then washed twice with PBS, and the dispersions containing the nanoparticles diluted in supplemented RPMI 1640 culture medium were added, obtaining final concentrations of 400 and 12.5 μg/mL. The cells were incubated with the nanoparticle dispersions for 24, 48, and 72 h at 37 °C and 5% CO_2_, and the treatment was carried out every 24 h. To calculate the wound area, the wounds were photographed under an optical microscope with a 40× objective, and the area was measured using ImageJ software v.1.8.0 (NIH, MA, USA).

### 2.7 Digestion of Lev-NPs, Chi-NPs, and Fuc-NPs

The nanoparticles were exposed to simulated gastrointestinal fluids to assess the digestion of the nanosystems. To do this, solutions simulating salivary (SSF), gastric (SGF), and intestinal (SIF) fluids were prepared following the updated INFOGEST guidelines.^21^

#### 2.7.1 Simulated Salivary Digestion

In the SSF (pH 7.0), an electrolyte stock solution was used according to the salt concentration determined by INFOGEST^21^ plus salivary amylase (1 mg/mL), the samples were kept in contact with SSF for 30 min, and aliquots were collected at 5, 15, and 30 min to evaluate the size, PDI, and surface charge on the Zetasizer nano ZS90 (Malvern Instruments, USA).

#### 2.7.2 Simulated Gastric Digestion

To study digestion in simulated gastric fluid, half of the SSF solution containing the nanoparticles was added to the SGF solution (ratio 1:1 v/v SSF:SGF) containing the salt solution, pepsin solution (0.0032 g/mL), and lipase solution (3.2 mg/mL). The pH of the final solution was 3.0. The nanoparticles were exposed for 2 h, and aliquots were collected every 30 min for subsequent analysis on the Zetasizer nano ZS90 (Malvern Instruments, USA).

#### 2.7.3 Simulated Intestinal Digestion

To evaluate digestion in the simulated intestinal fluid, after 2 h of exposure to SGF fluid, half of the SGF solution containing nanoparticles was mixed with the SIF solution (SGF:SIF ratio 1:1 v/v) composed of salt solution at the final concentration recommended by INFOGEST^21^ plus pancreatic trypsin (2.25 mg/mL) and bile salts (12 mg/mL), pH 7.0. The nanoparticles were exposed to SIF for 2 h, and aliquots were collected every 30 min for analysis on the Zetasizer nano ZS90 (Malvern Instruments, USA).

## 3 Results and Discussion

### 3.1 Obtaining Lev-NPs, Chi-NPs, and Fuc-NPs

The nanoparticles obtained showed sizes in the nanometric range (Table 1), with Chi-NPs being the smallest with a size of around 123.2 ± 2.18 nm and Fuc-NPs the largest with a size of 294.5 ± 1.25 nm. These results are like those found in the literature: Silva et al.^10^ obtained levan nanoparticles of 244 ± 2 nm, and Bravo-Osuna et al.^22^ showed that chitosan nanoparticles were 181 ± 40 nm in size, which is close to the size obtained in this study. According to Cavalcanti et al.^12^ and Cavalcanti et al.,^23^ fucoidan nanoparticles had sizes of 316 ± 1.33 nm and 305 ± 6.4 nm, like those in this study.

**Table 1:** Physicochemical Characterization of Nanoparticles

**Sample**	**Size (nm)**	**PDI**	**Zeta potential (mV)**
Lev-NPs	244.8 ± 2.44	0.07 ± 0.04	+3.93 ± 0.08
Chi-NPs	123.2 ± 2.18	0.19 ± 0.01	+25.9 ± 3.94
Fuc-NPs	294.5 ± 1.25	0.03 ± 0.04	–52.7 ± 1.70

When it came to PDI, the values were below 0.2, showing monodisperse nanoparticles,^24^ which was expected since nanoparticles obtained by the AEP technique using IBCA tend to have PDI values below 0.2.^10–13^ Regarding the surface charge of the nanoparticles (Table 1), the measured zeta potential values were consistent with the chemical characteristics of the polysaccharides used as surface-forming materials. Lev-NPs exhibited a near-neutral surface charge, which is consistent with the predominantly nonionic nature of levan, a fructan composed mainly of β-(2→6)-linked fructose units with β-(2→1)-linked branching and lacking ionizable groups under the experimental conditions.^16^ This profile agrees with the findings reported by Silva et al.^10^ In contrast, Chi-NPs displayed a positive surface charge, which can be attributed to the protonation of the primary amino groups (−NH_2_) of the glucosamine residues in chitosan, generating positively charged −NH_3_^+^ groups under acidic or mildly acidic conditions.^16,22,25^ Conversely, Fuc-NPs exhibited a negative surface charge, primarily associated with the sulfate ester groups (−OSO_3_^–^) present along the fucoidan backbone. The degree and distribution of sulfation confer an overall anionic character to fucoidan, favoring the development of negatively charged nanoparticle surfaces.^11–13,16^ Thus, the distinct zeta potential profiles of the three formulations reflect the chemical composition and ionizable functional groups of the respective polysaccharides, which may influence their colloidal stability and interactions with biological components.

#### 3.1.1 Morphological Aspects of Nanoparticles

TEM showed spherical nanoparticles with a homogeneous distribution (Figure 1), results that are in line with those presented in the PDI. It is also possible to see the presence of the corona formed by the polysaccharides, which is more evident in the images of the Lev-NPs (Figure 1A) and the Fuc-NPs (Figure 1C); as the Chi-NPs are very small, it was not possible to see the presence of the corona. According to the study by Lira et al.,^11^ the nanoparticles obtained by the AEP technique have a spherical shape and form nanoparticles with core–corona characteristics.

**Figure 1:**
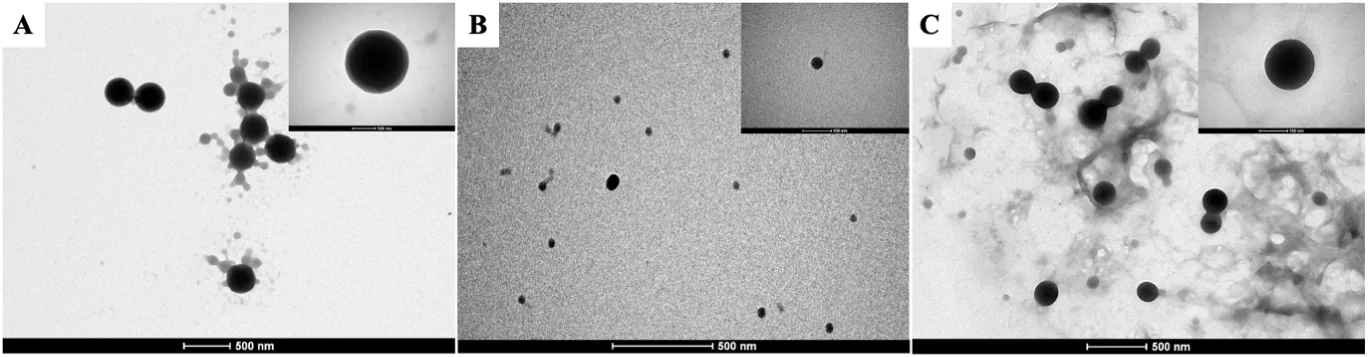
Morphological aspects of Lev-NPs (A), Chi-NPs (B), and Fuc-NPs (C) observed by TEM.

### 3.2 Blood Compatibility Studies

#### 3.2.1 Hemolytic Toxicity Assay

The intravenous route is one of the main routes of administration for nanocarriers, and determining the safety of administering these nanoparticles in the blood is essential. Intravenous administration of nanoparticles can result in high blood production at the site of administration, making it necessary to study the blood biocompatibility of these nanocarriers and ensure their safety *in vivo*.^26,27^ The induction of hemolysis by nanoparticles can result in anemia, hypertension, and renal toxicity, among other clinical situations.^26,28^ The degree of hemolysis of the nanoparticles in healthy human blood samples was evaluated.

In the present study, Lev-NPs, Chi-NPs, and Fuc-NPs (Figure 2) did not induce detectable hemolysis of red blood cells at any of the concentrations evaluated. This finding was supported by the macroscopic appearance of the samples, which remained comparable to the negative control and markedly different from the positive control. These results suggest that the nanoparticle formulations, as tested, displayed a favorable hemocompatibility profile. Levan is a biocompatible polysaccharide that has previously been investigated for applications in blood-contacting systems, including as a plasma volume expander.^29,30^ Similarly, Patel and Agrawal^31^ reported that levan-based micelles with a mean particle size of 153.1 ± 2.31 nm did not exhibit detectable hematological toxicity. These observations are consistent with the previously reported biocompatibility of levan-based systems; however, the hemolytic profile observed here reflects the behavior of the nanoparticle formulations, which may differ from that of the free polysaccharide due to nanoscale presentation and interactions with the PIBCA matrix.

**Figure 2:**
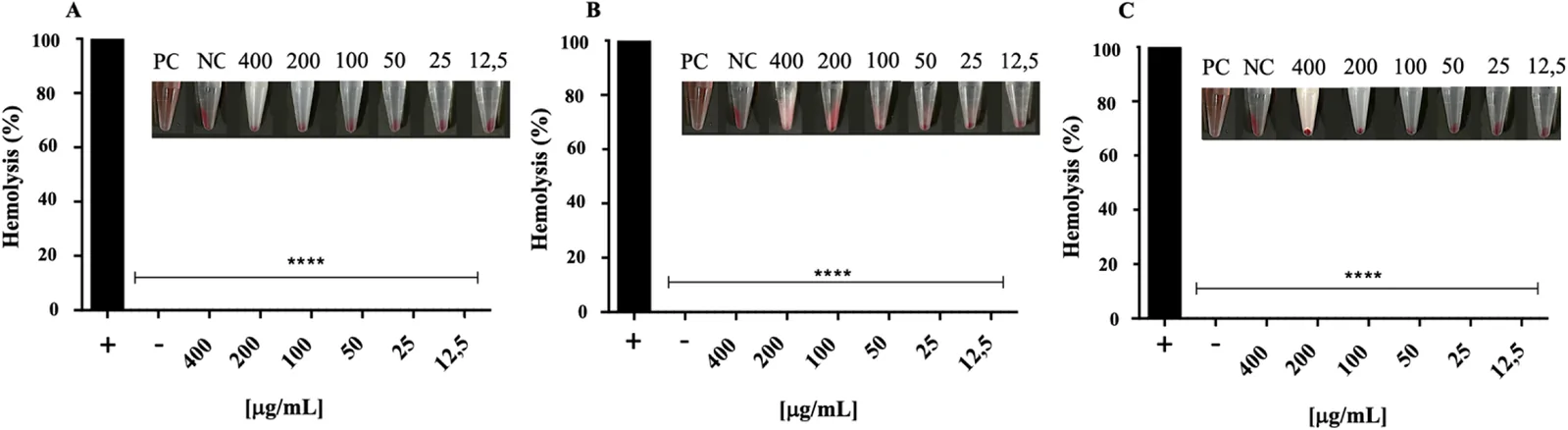
Hemolysis profile of Lev-NPs (A), Chi-NPs (B), and Fuc-NPs (C). PC = Positive control; NC = negative control. **** p-value (< 0.001).

Regarding Chi-NPs, Jesus et al.^32^ evaluated the degree of hemolysis of chitosan nanoparticles and found that at concentrations of 0.1, 1, and 2 mg/mL, they were not toxic to red blood cells. Low hematological toxicity was also demonstrated in the study by Gomathi et al.^33^ for chitosan nanoparticles, with these results surpassing those observed in this study with Chi-NPs (Figure 2B). As for the Fuc-NPs (Figure 2C), we confirmed the low hematological toxicity of these nanoparticles, as reported in a previous study by Cavalcanti et al.^12^ In view of this, Lev-NPs, Chi-NPs, and Fuc-NPs at the concentrations tested showed no hemolytic activity, with 0% hemolysis, proving to be promising nanoparticles.

#### 3.2.2 Coagulation Studies

Nanosystems can interact with one or more components of the coagulation cascade, potentially altering hemostatic homeostasis. Prothrombin time (pT) and activated partial thromboplastin time (aPTT) are conventional coagulation assays used to assess the activity of clotting factors involved predominantly in the extrinsic and intrinsic pathways, respectively, using decalcified plasma samples.^34–36^

As shown in Figure 3, Lev-NPs (Figure 3A) and Chi-NPs (Figure 3B) exhibited a predominantly procoagulant profile, as evidenced by shorter pT values than those observed for the control at all concentrations evaluated. These findings are consistent with trends reported for systems containing these polysaccharides, although the behavior observed here likely reflects nanoparticle-specific characteristics. Osman, Lin, and Hwang^37^ reported enhanced blood coagulation in levan-based hydrogels. Jesus et al.^32^ demonstrated in their study results like those presented in this study, using chitosan nanoparticles ranging in size from 127 ± 5 nm to 292 ± 52 nm, with a pT of approximately 11–14 s. The observed profiles are compatible with known biological tendencies of the respective polysaccharides; however, the mechanisms underlying the nanoparticle behavior cannot be inferred solely from the properties of the free polymers. Surface density, orientation, and nanoparticle architecture may also contribute to the coagulation effects observed.

**Figure 3:**
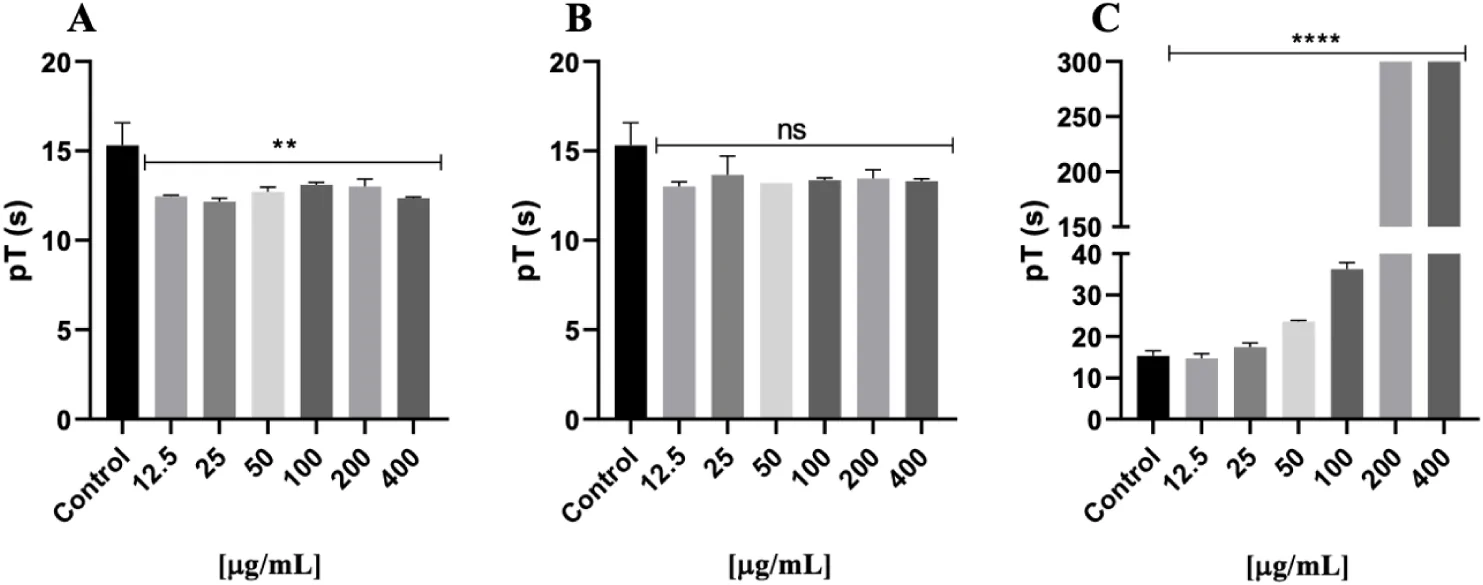
Prothrombin time of Lev-NPs (A), Chi-NPs (B), and Fuc-NPs (C) at different concentrations. **** p-value (< 0.001); ** p-value (0.0012); ns (>9999).

In contrast, Fuc-NPs (Figure 3C) exhibited a concentration-dependent anticoagulant profile. At the lowest concentration evaluated, no apparent anticoagulant effect was observed, whereas concentrations above 25 μg/mL resulted in prolonged pT values, indicating inhibition of coagulation through the extrinsic pathway. Previous studies have demonstrated that fucoidan possesses both anticoagulant and antithrombotic activities, supporting its potential as a bioactive polysaccharide for modulating hemostasis without necessarily inducing a pronounced bleeding response.^39–41^ In general, these results may indicate that the nature of the polysaccharide coating can influence the aggregation profile of the nanoparticles and may also be related to the specific physicochemical characteristics of each formulation.

The aPTT results (Figure 4) further support the pT findings, confirming the procoagulant activity of Lev-NPs and Chi-NPs and the anticoagulant activity of Fuc-NPs. Notably, the coagulation response of Lev-NPs and Chi-NPs appeared to be concentration-dependent, with an apparent anticoagulant effect observed at 50 μg/mL for Lev-NPs (Figure 4A) and at 100 and 200 μg/mL for Chi-NPs (Figure 4B). Although the concentration-dependent effects observed for Chi-NPs resemble trends reported for chitosan oligosaccharides,^42^ the nanoparticle context introduces additional variables—such as surface presentation and protein adsorption—that may modulate the intrinsic activity of the polysaccharide. According to Jesus et al.,^32^ the concentration of chitosan nanoparticles influences the prolongation of aPTT, noting that only the 80% chitosan nanoparticles at a concentration of 1 mg/mL showed a prolongation of aPTT, unlike the 93% chitosan nanoparticles. These results are consistent with those presented for Chi-NPs (Figure 4B), in which only the intermediate concentrations showed a prolongation of aPTT.

**Figure 4:**
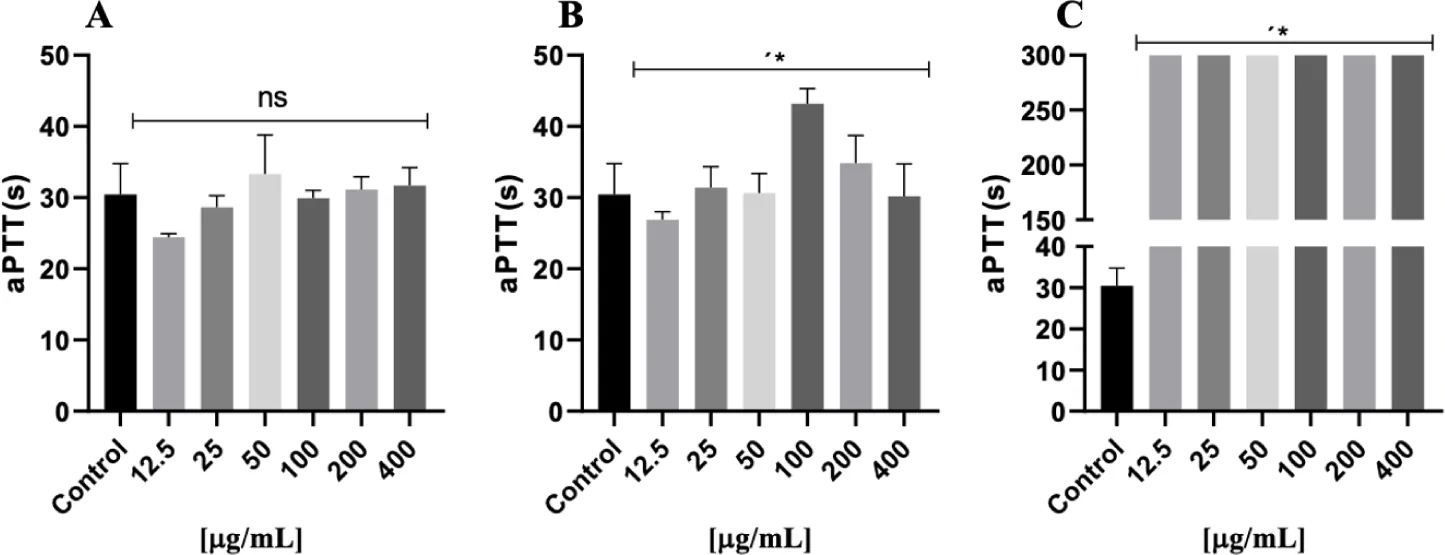
Activated partial thromboplastin time of Lev-NPs (A), Chi-NPs (B), and Fuc-NPs (C). * p-value (< 0.05); ns (>9999).

Similarly, Erginer et al.^43^ demonstrated that sulfated levan exhibits concentration-dependent anticoagulant activity through an intrinsic pathway. Interestingly, the present findings suggest that the sulfation state of levan may play a critical role in determining its coagulation profile. While nonsulfated levan exhibited predominantly procoagulant properties, sulfation of the polymer shifted its activity toward an anticoagulant profile, resembling the behavior reported for sulfated polysaccharides such as fucoidan and heparin.^44^ These comparisons suggest possible mechanisms but do not demonstrate them for nanoparticle formulations. The behavior of Lev-NPs may be influenced not only by levan chemistry but also by nanoparticle size, charge, and corona formation.

The Fuc-NP results further demonstrated a differential effect depending on the coagulation pathway evaluated. Fuc-NPs exhibited concentration-dependent anticoagulant activity in the extrinsic pathway (Figure 3C), whereas their anticoagulant effect in the intrinsic pathway was not significantly dependent on the concentration (Figure 4C). These findings are consistent with previous studies reporting that fucoidan interferes with coagulation by inhibiting Factor Xa formation and/or activity within the intrinsic coagulation pathway.^45–48^ Taken together, these results suggest that the nature of the polysaccharide coating and its structural features can determine the interaction of nanoparticles with distinct components of the coagulation cascade, thereby modulating their overall hemocompatibility profiles.

##### 3.2.2.1 Coagulation Kinetics

In the coagulation kinetics study, it can be seen in Figure 5A that Lev-NPs induced clot formation within the first few minutes of incubation with blood, especially at the highest concentration of Lev-NPs (Figure 5A, **red line**). These results are in line with those presented in the coagulation study (Section 3.2.2) and with the literature.^37^ The Chi-NPs (Figure 5B) showed their coagulant activity, with clot formation in the first few minutes but evident at the highest concentration (Figure 5B, **red line**), and it is possible to observe the presence of the clot in Figure S1f. The results were in line with the coagulation results and with the study by Wang et al.,^38^ showing that chitosan has good coagulating activity.

**Figure 5:**
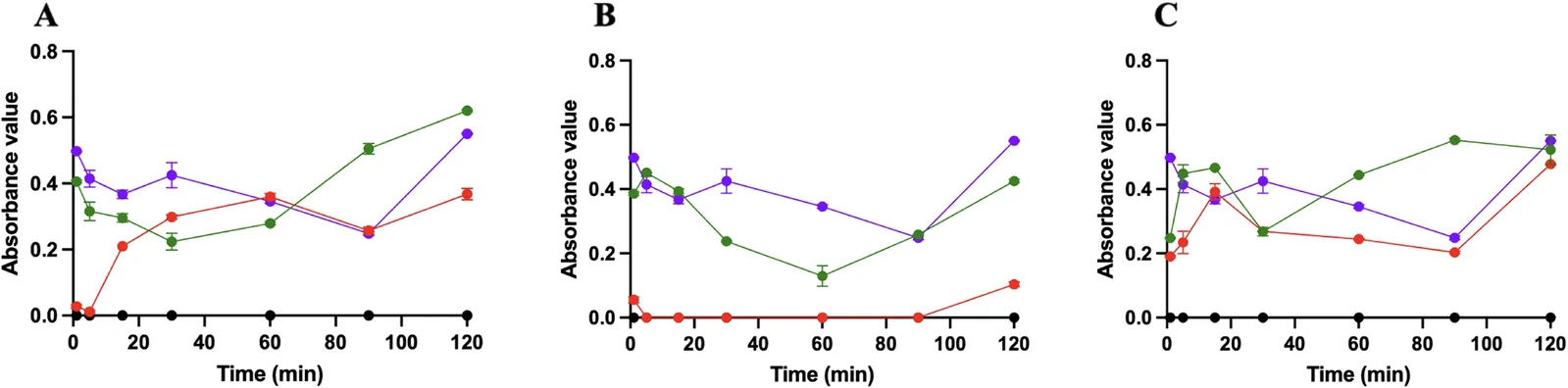
Coagulation kinetics profile of Lev-NPs (A), Chi-NPs (B), and Fuc-NPs (C). The curves represent the time-dependent absorbance changes for different experimental conditions: positive control (**purple line**), negative control (**black line**), low nanoparticle concentration (**dark green line**), and high nanoparticle concentration (**red line**). The trend of decreasing absorbance observed for higher nanoparticle concentrations suggests enhanced coagulation activity.

The Fuc-NPs (Figure 5C) appear to delay clot formation with a clotting kinetics profile that is somewhat similar to the control at higher concentrations (Figure 5C, **red line**) and at lower concentrations (Figure 5C, **dark green line**), it delays clotting, becoming more evident at 60 and 90 min, results that are consistent with the clotting study and corroborate the studies by Silva et al.,^39^ Anisha et al.,^40^ and Wang et al.^41^

#### 3.2.3 Evaluation of Clot Formation

During the coagulation process, platelets play an important role by adhering to the damaged area of the vessel and releasing substances that activate coagulation factors. In this process, platelets lose their rounded shape and become spiculated, interacting with blood proteins to form fibrin filaments containing red blood cells and more platelets, thus producing a stable clot.^49–51^ In the images obtained using fluorescence microscopy, we can see the stages in the control (Figure 6, **black box**) evident in the images of the group without fluorescence (blank), in which in the first 5 min (Figure 6A, **black box**), only platelets are observed, at 15 min (Figure 6B, **black box**), platelets are observed with some in a spiculated format, at 60 min (Figure 6C, **black box**), it is possible to see the presence of small clots, platelets in a round and spiculated format, and finally, at 120 min (Figure 6D, **black box**), there is the presence of larger clots with several platelets in a spiculated format. The presence of fluorescence in the Alexa 488, DAPI, and rhodamine filters may be related to the fibrin present in the coagulum, since fibrin seems to show autofluorescence in these three channels.^52^

**Figure 6:**
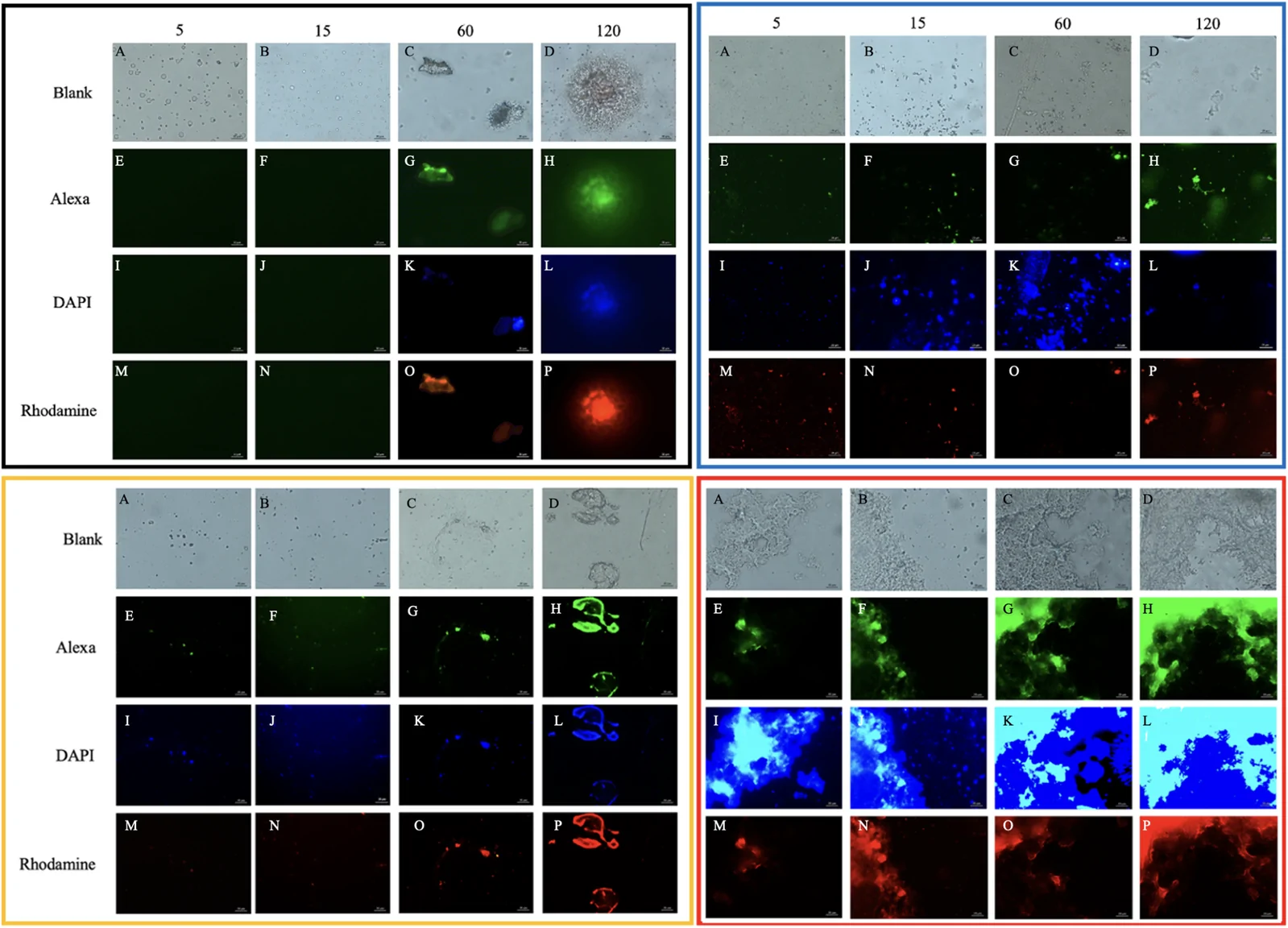
Cellular uptake and localization of the fluorescent marker (oncoA) following exposure to nanoparticles. Representative fluorescence micrographs showing the distribution of the fluorescent marker in samples incubated with buffer (control, **black box**), Lev-NPs (**gold box**), Fuc-NPs (**blue box**), and Chi-NPs (**red box**) for 5, 15, 60, and 120 min. The fluorescence patterns observed over time suggest changes in the distribution and accumulation of the fluorescent marker associated with the nanoparticles. Panels A–P correspond to the different nanoparticle formulations and incubation times. Images were acquired using Alexa 488, DAPI, and rhodamine fluorescence channels with a 20× objective.

The results showed that Lev-NPs (Figure 6, **gold box**) induced morphological changes in platelets within the first 5 min (Figure 6A, **gold box**), with visible clot formation at 60 min (Figure 6C, **gold box**), demonstrating their coagulation activity, consistent with the other results. Fluorescent labeling of oncoA using Alexa 488, DAPI, and rhodamine filters^23^ shows the interaction of Lev-NPs with platelets—evident at 5 and 15 min—as well as the presence of fluorescent nanoparticles within the formed clots.

In contrast, Chi-NPs (Figure 6, **red box**) were associated with earlier clot formation, observed as early as the first 5 min (Figure 6A, **red box**). The presence of the nanoparticles in the clot is evident due to the high fluorescence intensity. These findings are consistent with the reported procoagulant properties of chitosan, a natural polymer previously described as capable of promoting platelet-related coagulation processes through mechanisms involving the expression of integrin αIIbβ3 and P-selectin.^53,54^ However, since the present analysis was based primarily on morphological observations, the occurrence of platelet activation cannot be conclusively determined without specific functional or molecular markers of platelet activation.

For the Fuc-NPs (Figure 6, **blue box**), morphological changes in platelets were observed within the first 5 min (Figure 6A, **blue box**) compared to the control, accompanied by the appearance of fibrin. After 60 min (Figure 6C, **blue box**), small clot-like structures were observed, which remained detectable at 120 min (Figure 6D, **blue box**), when a greater abundance of fibrin also became evident. Fluorescence microscopy using Alexa Fluor 488, DAPI, and rhodamine filters revealed a concentration of Fuc-NPs associated with platelets and, subsequently, with fibrin filaments, with some nanoparticles appearing to be trapped within the clot-like structures.

These findings suggest that Fuc-NPs are associated with time-dependent morphological changes in platelets and with the formation of fibrin- and clot-like structures. The interaction of Fuc-NPs with fibrin and their apparent entrapment within clot-like structures may reflect their involvement in the coagulation process and should be considered in the context of the previously reported anticoagulant properties of Fuc-NPs, including their ability to interfere with coagulation factors such as factor Xa.

Previous studies have reported that fucoidan may interact with platelets through Src- and Syk-dependent signaling pathways and may act as an agonist of the C2-type lectin receptor (CLEC-2).^55,56^ However, the involvement of these pathways in the morphological changes observed in the present study cannot be confirmed solely on the basis of fluorescence microscopy. Further analyses using specific markers of platelet activation and functional assays would be necessary to determine whether the observed morphological changes are associated with platelet activation.

#### 3.2.4 Platelet Aggregation

The results of the platelet aggregation study are consistent with the other coagulation and clot formation studies, where it is possible to observe the presence of clot formation in the Lev-NPs sample (Figure 7D) and a well-formed clot in the Chi-NPs sample (Figure 7C).^37,38^ Jesus et al.^32^ pointed out that chitosan nanoparticles were able to induce platelet aggregation at higher concentrations of chitosan. On the other hand, the interaction of Fuc-NPs with platelets (Figure 7B) can be seen, with the absence of a clot.^39–41^ The SEM results visually reinforce the clotting effects of Lev-NPs and Chi-NPs and the anticoagulant effect of Fuc-NPs. While the platelet interactions observed may be compatible with the known properties of the free polysaccharides, the nanoscale organization of the coatings likely alters their biological presentation.

**Figure 7:**
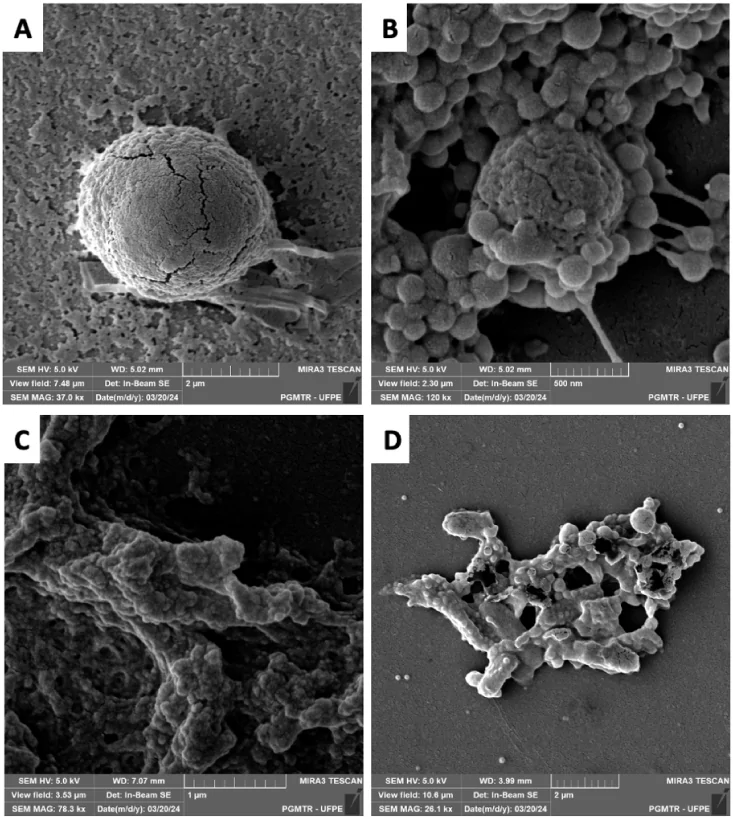
Macroscopic aspects of the samples after exposure to PBS (A), Fuc-NPs (B), Chi-NPs (C), and Lev-NPs (D).

### 3.3 Cytotoxicity of Nanoparticles

The results of the nanoparticle cytotoxicity study (Figure 8) revealed a toxicity profile for Lev-NPs (Figure 8A), Chi-NPs (Figure 8B), and Fuc-NPs (Figure 8C) with viability below 50% for the three cell lines. This cytotoxicity profile is noteworthy, particularly because the free polysaccharides levan, fucoidan, and chitosan are generally reported as noncytotoxic. However, nanoparticle formulations may behave differently from their isolated polysaccharide counterparts because of differences in surface presentation, density, and interactions with the PIBCA matrix.

**Figure 8:**
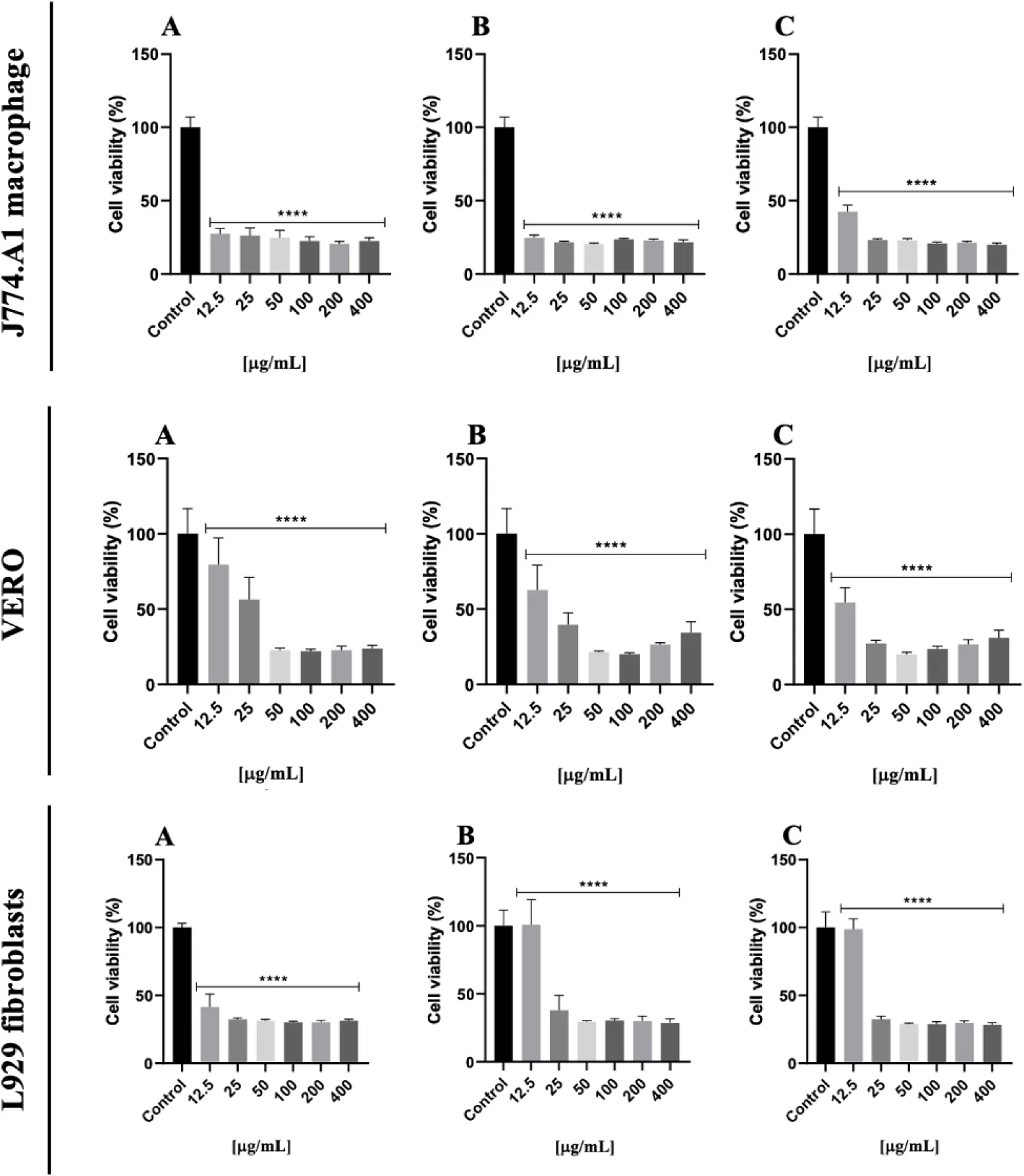
Cytotoxicity of Lev-NPs (A), Chi-NPs (B), and Fuc-NPs (C) on J774.A1 macrophage, VERO, and L929 fibroblast cell lines. **** p-value (<0.001).

Optical microscopy images showed preserved cellular morphology across most conditions even at higher concentrations (400 μg/mL); however, morphological integrity alone cannot confirm viability or exclude metabolic impairment. Figure S2 shows J774.A1 macrophages with preserved morphological features in the presence of Lev-NPs (Figure S2B), Chi-NPs (Figure S2C), and Fuc-NPs (Figure S2D) when exposed to both lower and higher concentrations.

Similar findings were observed in VERO cells (Figure S3), in which preserved cellular morphology was observed at both the lowest tested concentration (12.5 μg/mL) and the highest concentration (400 μg/mL) of Lev-NPs, Chi-NPs, and Fuc-NPs. Likewise, L929 fibroblasts (Figure S4) exhibited apparently preserved morphology across the tested samples, except for Lev-NPs at higher concentrations (Figure S4B, second row), where morphological alterations were more evident.

The discrepancy between the morphological observations and the MTT assay results is consistent with the possibility of assay interference or altered metabolic activity, but the present data do not allow us to determine the underlying mechanisms. Soares et al.,^57^ in a study evaluating ethyl cyanoacrylate nanoparticles coated with dextran or fucoidan, also reported a lack of correlation between MTT assay results and cellular morphology. Similarly, Sulheim et al.^58^ evaluated the cytotoxicity of poly­(alkyl cyanoacrylate) nanoparticles using three different approaches and observed differences between the results obtained with CellTiter-Glo and lactate dehydrogenase (LDH) assays and those obtained using the MTT assay, highlighting the challenges associated with the assessment of the cytotoxicity in nanosystem-based formulations.

Importantly, although the nanocarriers may interfere with cellular metabolic assays such as MTT, the observation of an apparently preserved cellular morphology by optical microscopy should also be interpreted with caution. Morphological integrity alone does not necessarily indicate the maintenance of cellular viability or metabolic activity. Therefore, the morphological analysis performed in the present study provides complementary qualitative information but cannot, by itself, be used to confirm cell viability. These findings underscore the importance of combining morphological assessment with complementary viability assays when evaluating the cytotoxicity of nanocarrier-based systems.

### 3.4 Cell Viability of Nanoparticles in PBMCs

MTT results (Figure 9) indicated viability below 50% for all formulations (Lev-NPs, Chi-NPs, and Fuc-NPs), whereas microscopic visualization suggested the presence of apparently viable cells (Figure S5). This inconsistency highlights the need for caution when interpreting the MTT data for nanoparticle systems.

**Figure 9:**
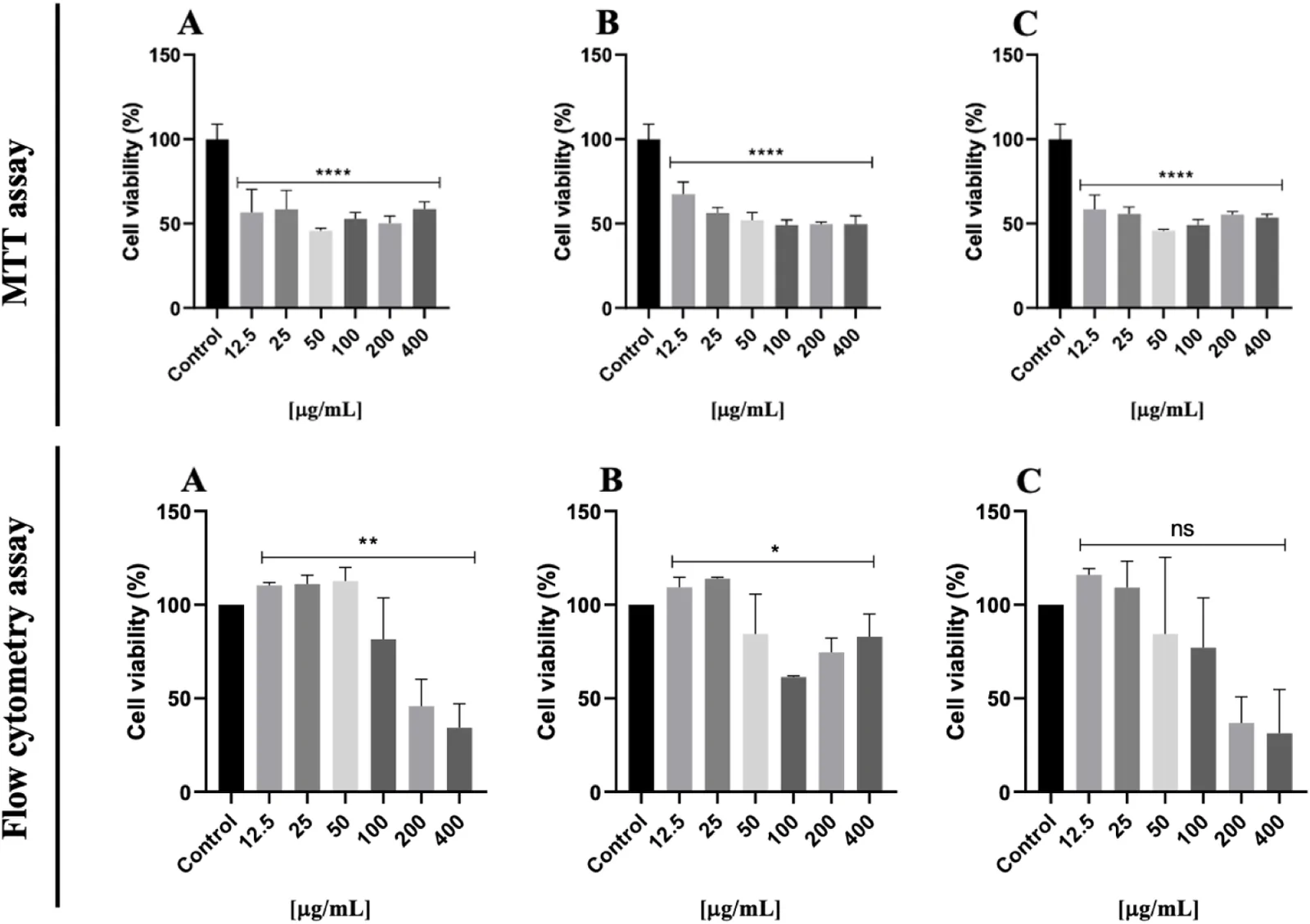
Cell viability of PBMCs after exposure to Lev-NPs (A), Chi-NPs (B), and Fuc-NPs (C).

Flow cytometry (Figure 9) indicated high cell viability, particularly at lower concentrations (from 50 to 12.5 μg/mL). When considered together with the MTT results, these findings are consistent with the possibility that the nanoparticles may affect MTT metabolism or formazan formation, but the mechanism cannot be determined from the present data.^57,58^

### 3.5 *In Vitro* Wound Healing

The wound-healing study on L929 fibroblast cells (Figures 10 and 11) shows that Fuc-NPs (Figure 10E, **lavender column**) and Lev-NPs (Figure 10E, **light blue column**) showed progressive wound closure with treatment every 24 h at lower concentrations when compared to the control (Figure 10, column A). The wound closure results for Chi-NPs (Figure 10E, **light orange column**) were more discrete with statistically similar results after the first 24 h of treatment.

**Figure 10:**
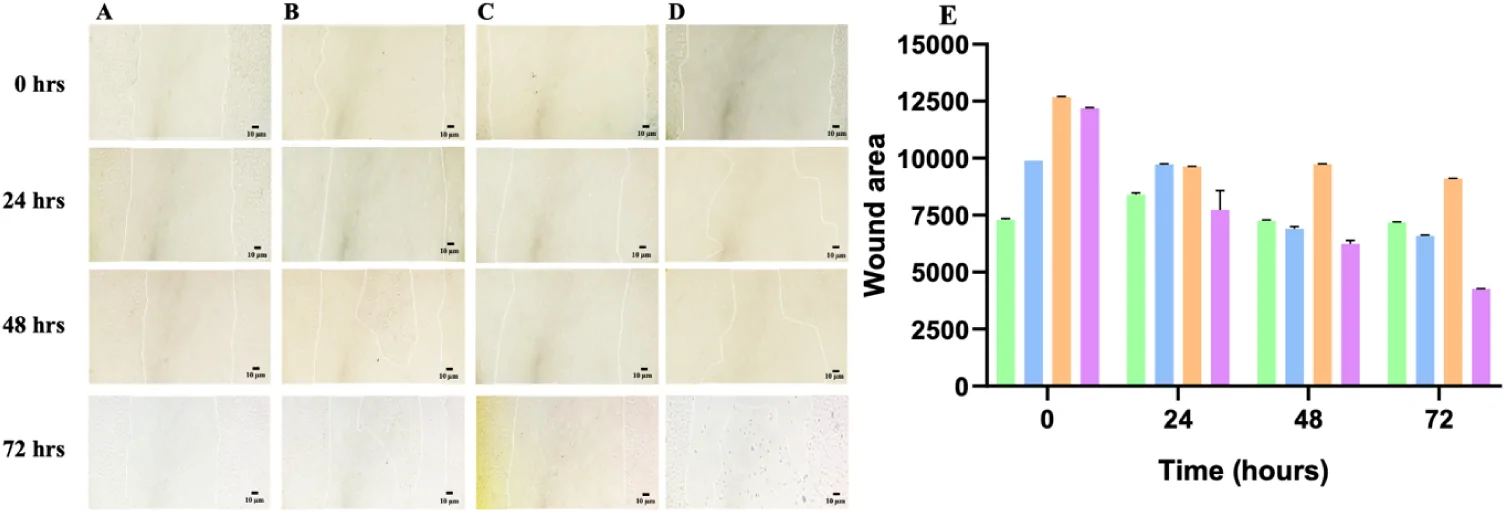
*In vitro* wound healing after treatment with culture medium (control) (column A), Lev-NPs (column B), Chi-NPs (column C), and Fuc-NPs (column D) after 0, 24, 48, and 72 h at a concentration of 12.5 μg/mL. Wound area closure profile over time (E): Control (**light green column**); Lev-NPs (**light blue column**); Chi-NPs (**light orange column**); Fuc-NPs (**lavender column**).

**Figure 11:**
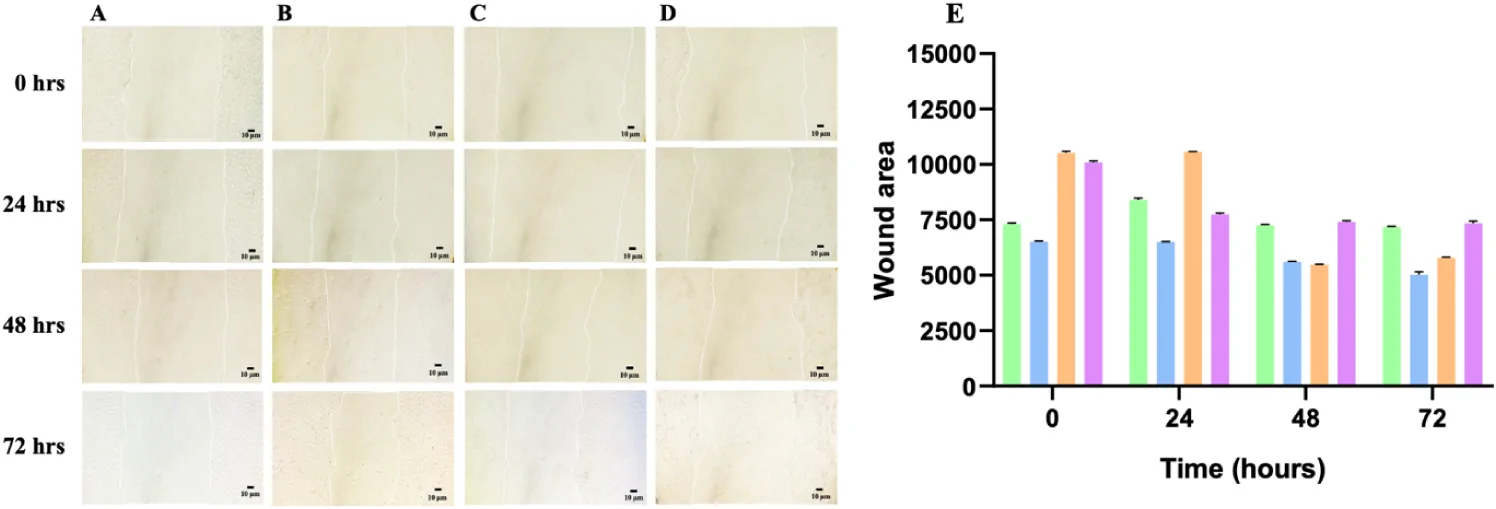
*In vitro* wound healing after treatment with culture medium (control) (column A), Lev-NPs (column B), Chi-NPs (column C), and Fuc-NPs (column D) after 0, 24, 48, and 72 h at a concentration of 400 μg/mL. Wound area closure profile over time (E): Control (**light green column**); Lev-NPs (**light blue column**); Chi-NPs (**light orange column**); Fuc-NPs (**lavender column**).

At concentrations of 400 μg/mL (Figure 11), all the nanoparticles showed a good wound healing profile, with Chi-NPs standing out (Figure 11E, **light orange column**), which at higher concentrations showed a better wound area closure profile, with a slight evolution toward Lev-NPs (Figure 11E, **light blue column**) and Fuc-NPs (Figure 11E, **lavender column**). In contrast to the results of the 12.5 μg/mL concentration, the Chi-NPs (Figure 11, column C) showed a better healing profile with very evident results after 48 h of treatment. As for Fuc-NPs (Figures 10 and 11, column D), lower concentrations seem to have greater efficacy in wound healing in vitro, while Lev-NPs (Figures 10 and 11, column B) showed a good healing response at both higher (400 μg/mL) and lower (12 μg/mL) concentrations.

According to Osman et al.,^37^ levan hydrogels promoted wound healing *in vitro* with NIH 3T3 and HaCaT cells, showing the benefits of levan in wound healing. Not dissimilarly, Yu et al.^59^ highlighted the benefits of carboxymethyl chitosan hydrogels in wound healing by promoting the proliferation of granulation tissue and re-epithelialization, increasing the viability and migration of L929 cells; similar results were also presented by Behera et al.^60^ Fucoidan, on the other hand, does not seem to be widely used in the development of healing products, as it is usually associated with other polysaccharides (chitosan and alginate), as in the study by Nakamura et al.^61^ and Murakami et al.^62^ Despite this, Fuc-NPs can be used to deliver drugs that contribute to wound healing, and as shown in this study, Fuc-NPs appear to promote wound healing at a concentration of 12.5 μg/mL.

### 3.6 Digestion of Lev-NPs, Chi-NPs, and Fuc-NPs

After oral ingestion, nanoparticles undergo various processes, such as pH changes and biochemical components that are part of food digestion. These processes can alter the physicochemical characteristics of these nanosystems and compromise their function in delivering drugs to target sites.^63–66^ Understanding the behavior of nanocarriers during the digestion process is essential for proposing routes of administration and/or defining strategies that favor the delivery of nanocarriers and their absorption at the appropriate site (absorption in the oral cavity, stomach, or intestines).^67,68^

Figure 12 shows the results of the *in vitro* digestion process of the nanoparticles. It can be seen in Figure 12A that the Lev-NPs (Figure 12A, **orange line**) and Fuc-NPs (Figure 12A, **pink line**) did not show significant changes in size in the three phases of digestion (SSF, SGF, and SIF). The results are consistent with the PDI, which show monodispersions (Figure 12B, **orange** and **purple** lines). With different results, the Chi-NPs (Figure 12A, **purple line**) showed a change in size, increasing their size in the SSF from 123.2 ± 2.18 nm to 653.9 ± 534.41 nm, showing the instability of these nanoparticles in the first 30 min of digestion. The instability of the Chi-NPs is evident in the PDI results (Figure 12B, **purple line**), showing the presence of heterogeneous dispersion.

**Figure 12:**
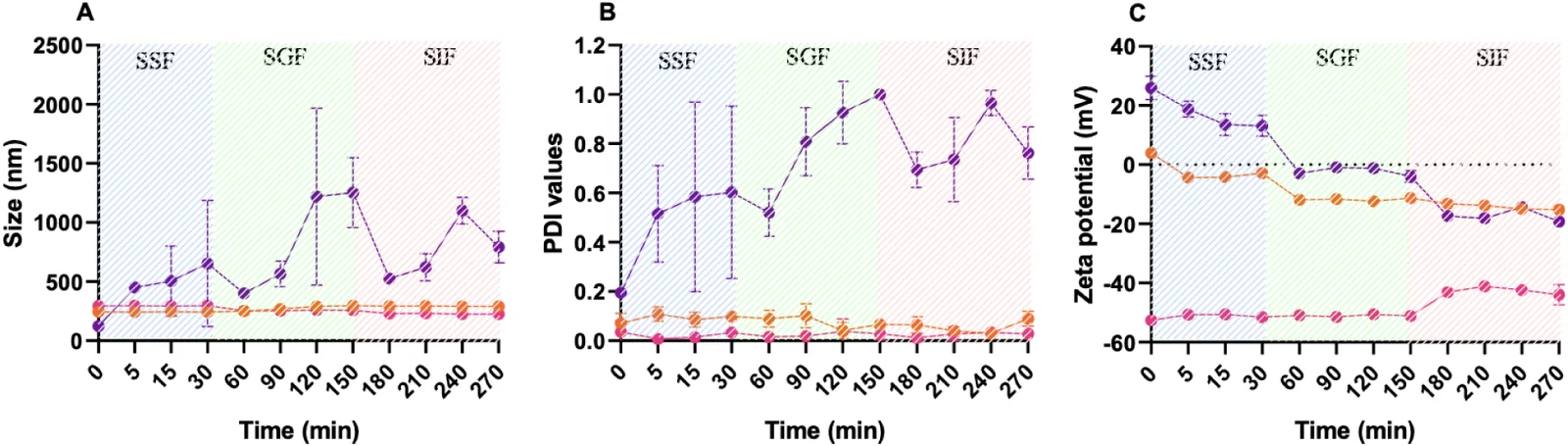
Digestion of Lev-NPs (**orange line**), Chi-NPs (**purple line**), and Fuc-NPs (**pink line**). Results of size (A), PDI (B), and zeta potential (C). SSF = Simulated Salivary Fluid; SGF = Simulated Gastric Fluid; SIF = Simulated Intestinal Fluid.

According to the results of the surface charge of the nanoparticles (Figure 12C), the Fuc-NPs (Figure 12C, **pink line**) maintained their surface charge during the journey in the SSF and SGF, but in the SIF, there was a slight change from −52.7 ± 1.70 mV to −44.0 ± 3.46 mV; this change may be related to the interaction of the Fuc-NPs with pancreatic trypsin and/or bile salts. Koh et al.^69^ reported that fucoidan can modulate enzymatic activity during intestinal digestion, which may partially align with the surface charge changes observed here, but additional studies are required to confirm whether similar interactions occur in nanoparticle form.

As for the Lev-NPs (Figure 12C, **orange line**), there was a reduction in the surface charge in the first 5 min after the start of digestion, from 3.9 ± 0.08 mV to −4.4 ± 0.27 mV, remaining stable throughout the 30 min of SSF. In SGF, there was another change in the surface charge of the Lev-NPs to −11.9 ± 0.51 mV, remaining stable throughout the SGF and SIF phases. Xu et al.^70^ reported that levan undergoes limited degradation in salivary conditions but experiences molecular weight reduction under gastric acidity, which is consistent with the gradual changes in surface charge observed in this study. However, because zeta potential reflects multiple factors—including ionic strength and adsorption of digestive components—these results should be interpreted cautiously.

Chi-NPs (Figure 12C, **purple line**) showed the most pronounced changes in surface charge. In SSF, the zeta potential decreased from +25.9 ± 3.94 mV to +18.8 ± 2.66 mV within 5 min, suggesting early interactions with salivary constituents. In SGF, the charge shifted to near-neutral values (−2.9 ± 0.13 mV), likely influenced by acidic pH and exposure to pepsin and lipase. Upon transition to SIF, the charge became strongly negative (−17.4 ± 0.15 mV), indicating substantial modification of the nanoparticle surface. These changes coincide with the observed increase in particle size and PDI, supporting the hypothesis that Chi-NPs undergo structural destabilization or aggregation during digestion. Similar charge inversion has been reported for chitosan-coated systems exposed to gastrointestinal conditions,^71^ although the specific contributions of pH, enzymes, and bile salts require further investigation.

Overall, the digestion results highlight that the three PIBCA formulations respond differently under gastrointestinal conditions. However, because particle size, surface charge, and polysaccharide density differ among formulations, the present data do not isolate polysaccharide identity as the sole factor driving these behaviors. Future studies incorporating controlled surface quantification and enzymatic interaction assays will be necessary to clarify the mechanisms underlying these digestion profiles.

## 4 Conclusion

This study investigated the in vitro behavior of three PIBCA nanoparticle formulations coated with different polysaccharides—levan, chitosan, and fucoidan—and demonstrated that the nature of the coating was associated with distinct biological responses, particularly regarding hematological compatibility, coagulation, cytotoxicity, wound healing, and gastrointestinal stability. Under the experimental conditions evaluated, none of the formulations showed evidence of hematological toxicity. Lev-NPs and Chi-NPs were associated with a procoagulant profile, whereas Fuc-NPs exhibited an anticoagulant profile. These findings suggest that the polysaccharide coating can substantially influence the biological behavior of PIBCA nanoparticles.

Regarding cytotoxicity, the tested nanoparticles showed no marked cytotoxic effects in the evaluated cell lines, particularly at lower concentrations, based primarily on an MTT assay. However, these findings should be interpreted with caution, as metabolic assays may be affected by nanoparticle-related interference, and preserved cellular morphology alone cannot confirm cell viability. Therefore, complementary assays are necessary to further characterize the cytotoxicity profiles of these formulations.

In the *in vitro* wound healing model, Lev-NPs and Chi-NPs were associated with a greater acceleration of the healing process compared with Fuc-NPs. Although this effect may be related, at least in part, to their observed procoagulant profile, the present data do not establish a causal relationship between the coagulation behavior and wound-healing performance. Regarding gastrointestinal stability, Lev-NPs and Fuc-NPs maintained their physicochemical characteristics throughout the simulated digestion process, whereas Chi-NPs exhibited a marked loss of stability within 5 min. These findings provide preliminary evidence that the polysaccharide coating may also influence the nanoparticle behavior under gastrointestinal conditions.

Overall, the results demonstrate that polysaccharide-coated PIBCA nanoparticles exhibit formulation-dependent *in vitro* properties that may be relevant to their further development. However, the present findings represent an initial *in vitro* characterization and are insufficient to establish the safety, clinical suitability, therapeutic efficacy, or optimal route of administration of any of the formulations. Consequently, the apparent suitability of Lev-NPs, Chi-NPs, and Fuc-NPs for specific administration routes should be considered as a hypothesis for further investigation rather than as a definitive indication.

A relevant limitation of the present study was the absence of a detailed evaluation of nanoparticle stability in biologically relevant media including phosphate-buffered saline (PBS), plasma, and cell culture media. Future studies should investigate the colloidal stability of these formulations under such conditions and determine whether changes in particle size, aggregation state, surface properties, or other physicochemical characteristics influence their biological interactions and performance. In addition, further investigations incorporating drug-loaded nanoparticles and appropriate *in vivo* models will be necessary to determine whether the biological behaviors observed *in vitro* translate into meaningful therapeutic effects under the complex physiological conditions of living organisms.

Taken together, the present study provides a comparative *in vitro* characterization of levan-, chitosan-, and fucoidan-coated PIBCA nanoparticles and emphasizes the importance of polymer selection in modulating the nanocarrier behavior. These findings may contribute to the rational design and future development of polysaccharide-coated PIBCA nanosystems while recognizing that substantial additional investigation is required before their safety, efficacy, and clinical applicability can be established.

## Supplementary Material



## Notes

Macroscopic appearance of the positive and negative controls and the formulations Fuc-NPs in high concentration and low concentration, Chi-NPs in high concentration and low concentration, and Lev-NPs in high concentration and low concentration (Figure S1); macroscopic aspects of J774.A1 macrophage cells and after exposure to Lev-NPs, Chi-NPs, and Fuc-NPs at lower and higher concentrations in an optical microscope (10× objective) (Figure S2); macroscopic aspects of VERO cells and after exposure to Lev-NPs, Chi-NPs, and Fuc-NPs at lower and higher concentrations in an optical microscope (10× objective) (Figure S3); macroscopic aspects of L929 fibroblast cells and after exposure to Lev-NPs, Chi-NPs, and Fuc-NPs at lower and higher concentrations in an optical microscope (10× objective) (Figure S4); and macroscopic aspects of PBMC cells and after exposure to Lev-NPs, Chi-NPs, and Fuc-NPs at lower and higher concentrations in an optical microscope (10× objective) (Figure S5) (PDF)
