# Supplementary material for: Biological Properties of Promising Poly(isobutyl cyanoacrylate) Nanoparticles Coated with Fucoidan, Levan, or Chitosan
Source: ACS Omega. 2026 Oct 9;11(41):63893–907. doi: 10.1021/acsomega.6c08464 (PMC13689201; doi:10.1021/acsomega.6c08464)
Supplement: Supplementary file 1 [file ao6c08464_si_001.pdf]

Biological properties of promising poly(isobutyl cyanoacrylate) nanoparticles coated with fucoidan, levan, or chitosan

Iago Dillion Lima Cavalcanti[1,2,6*], Andreza Tallyne de Aguiar Silva[1,2], Leandro Paes de Brito[3], Clarice Beatriz Gonçalves Silva[1], Fabrício Oliveira Souto[1], Romero Marcos Pedrosa Brandão Costa[4], Otília Deusdênia Loiola Pessoa[5], Eloisa Berbel Manaia[6], Gilles Ponchel[6], Nereide Stela Santos Magalhães[1], Mariane Cajubá de Britto Lira Nogueira[1,2*]

[1] Instituto Keizo-Asami (iLIKA), Universidade Federal de Pernambuco (UFPE), Recife, Brazil.

[2] Laboratório de Nanotecnologia, Biotecnologia e Cultura de Células, Centro Acadêmico de Vitória, Universidade Federal de Pernambuco (CAV/UFPE), Recife, Brazil.

[3] Laboratório de Avanços em Biotecnologia de Proteínas e Tecnologia de Bioativos, Universidade Federal Rural de Pernambuco (UFRPE), Recife, Brazil.

[4] Laboratório Integrado de Biotecnología Aplicada à Saúde, Universidade de Pernambuco (UPE), Recife, Brazil.

[5] Laboratório de Análise Fitoquímica de Plantas Medicinais II, Departamento de Química Orgânica e Inorgânica, Universidade Federal do Ceará, Fortaleza, Brazil.

[6] Faculté de Pharmacie Institut Galien Paris-Saclay - UMR-CNRS 8612, Université Paris-Saclay.

***Corresponding authors**:

Profa Dr[a] Mariane C. B. Lira Nogueira / Dr. Iago Dillion Lima Cavalcanti

Federal University of Pernambuco

Keizo-Asami Institute (iLIKA)

Av. Prof. Moraes Rego, 1235, Cidade Universitária, 50670-901, Recife-PE, Brazil

Tel: + 55 81 21268587 E-mail: mariane.lira@ufpe.br / iago.dillion@ufpe.br

Supporting Information

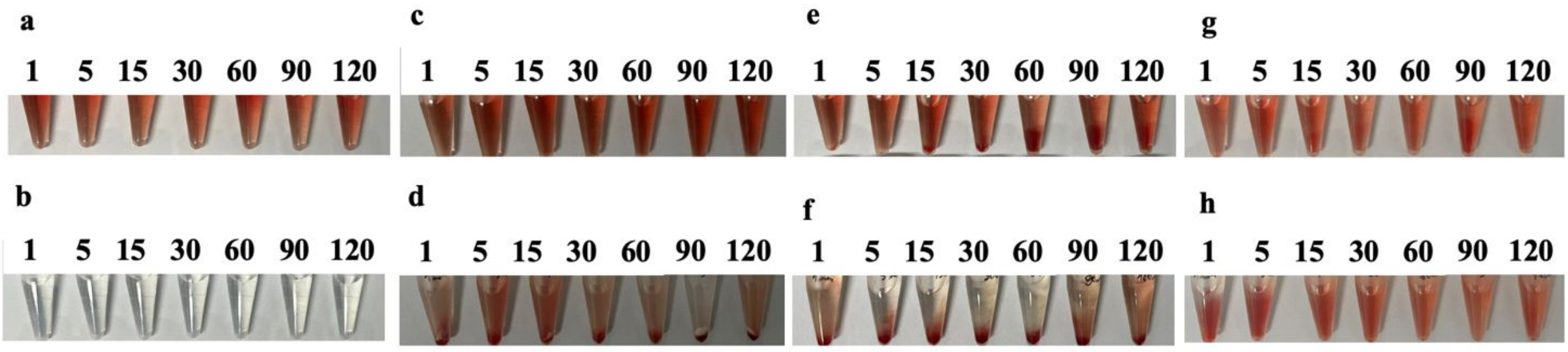

Figure S1. Macroscopic appearance of the positive (a) and negative (b) controls, and the formulations Fuc-NPs in high concentration (c) and low concentration (d), Chi-NPs in high concentration (e) and low concentration (f) and Lev-NPs in high concentration (g) and low concentration (h).

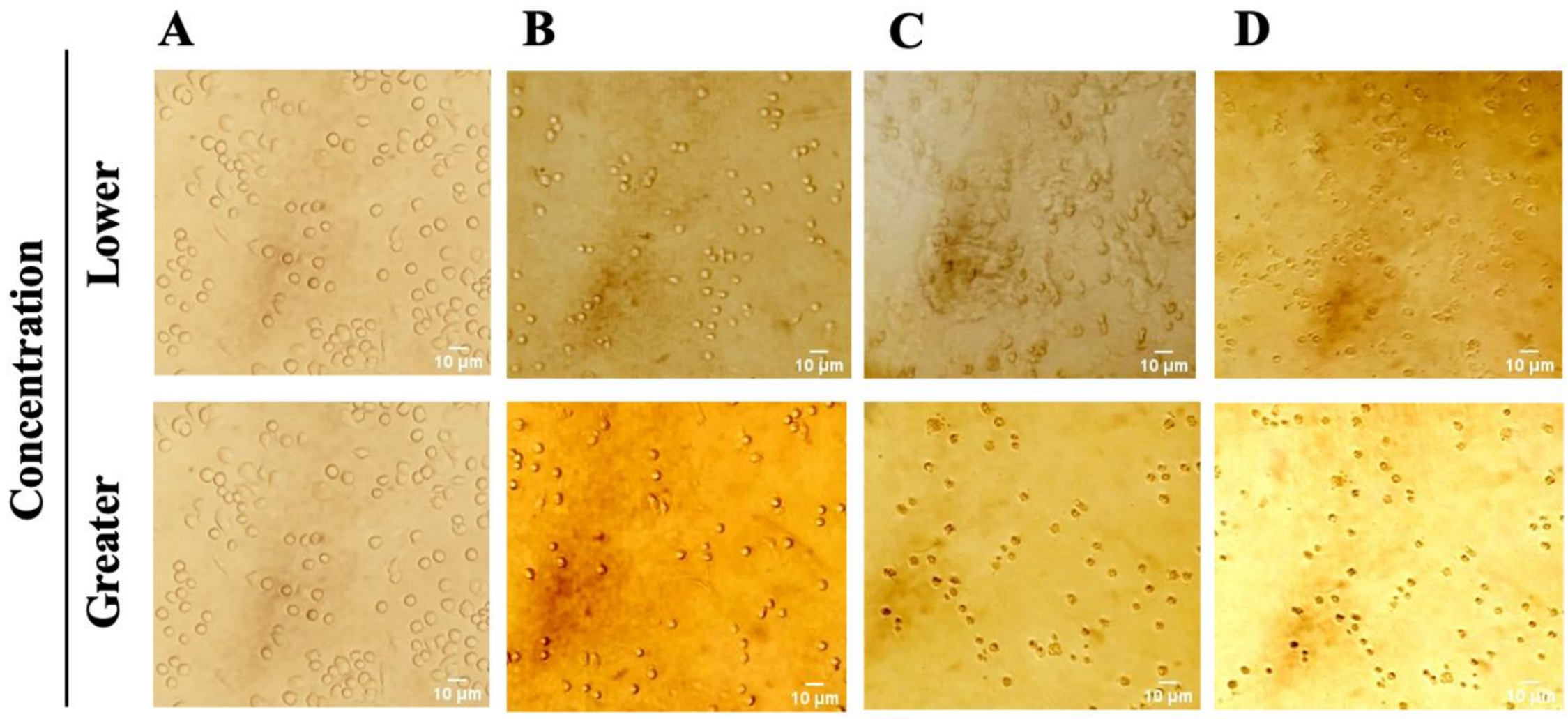

Figure S2. Macroscopic aspects of J774.A1 macrophage cells (A) and after exposure to Lev-NPs (B), Chi-NPs (C), and Fuc-NPs (D) at lower (first row) and greater (second row) concentrations in an optical microscope (10x objective).

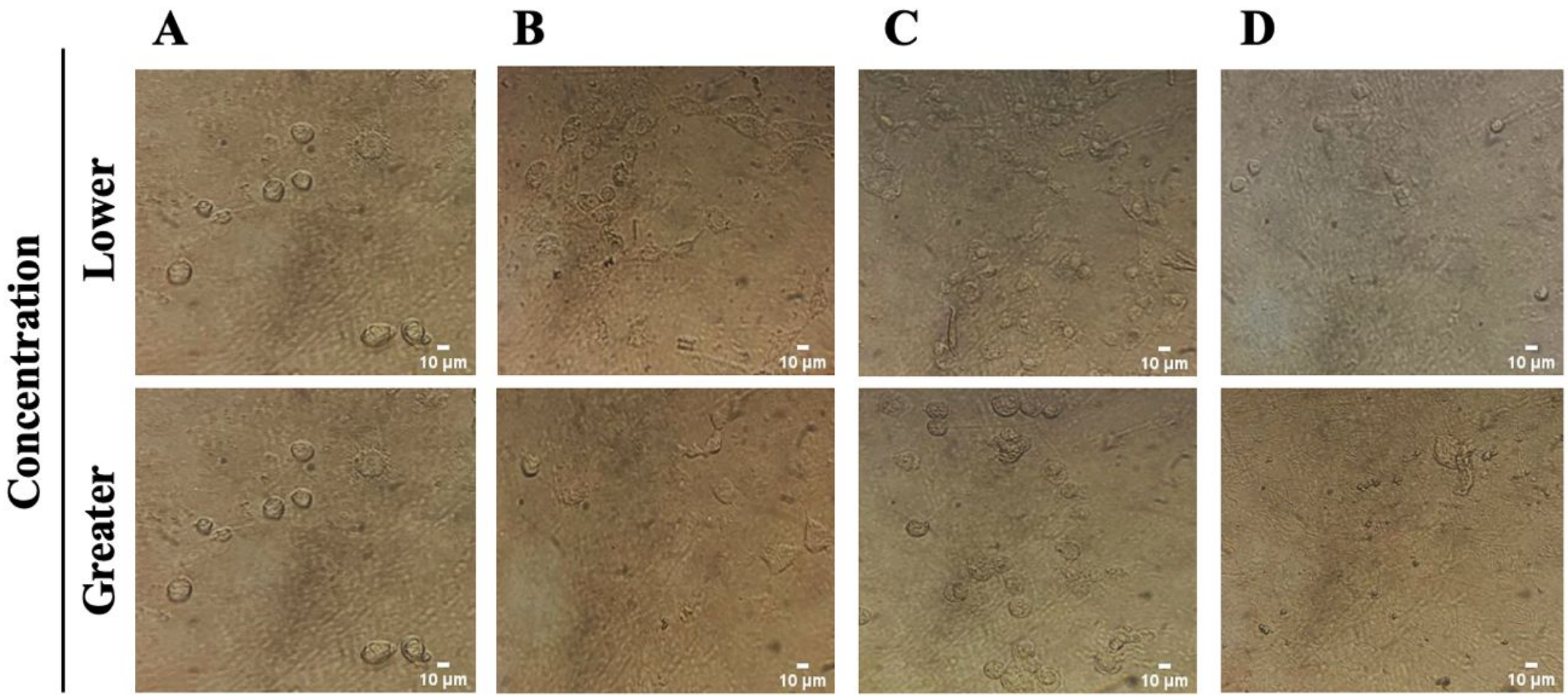

Figure S3. Macroscopic aspects of VERO cells (A) and after exposure to Lev-NPs (B), Chi-NPs (C), and Fuc-NPs (D) at lower (first row) and greater (second row) concentrations in an optical microscope (10x objective).

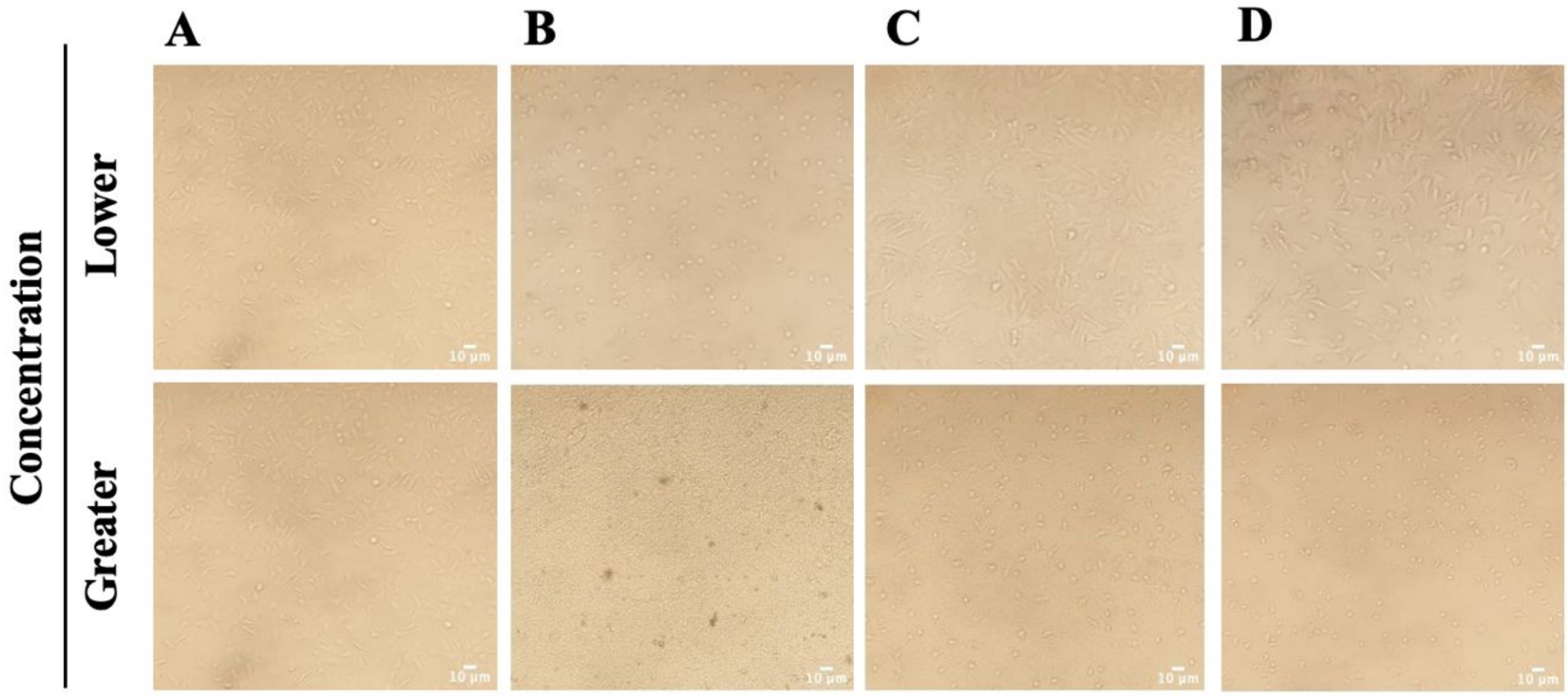

Figure S4. Macroscopic aspects of L929 fibroblasts cells (A) and after exposure to Lev-NPs (B), Chi-NPs (C), and Fuc-NPs (D) at lower (first row) and greater (second row) concentrations in an optical microscope (10x objective).

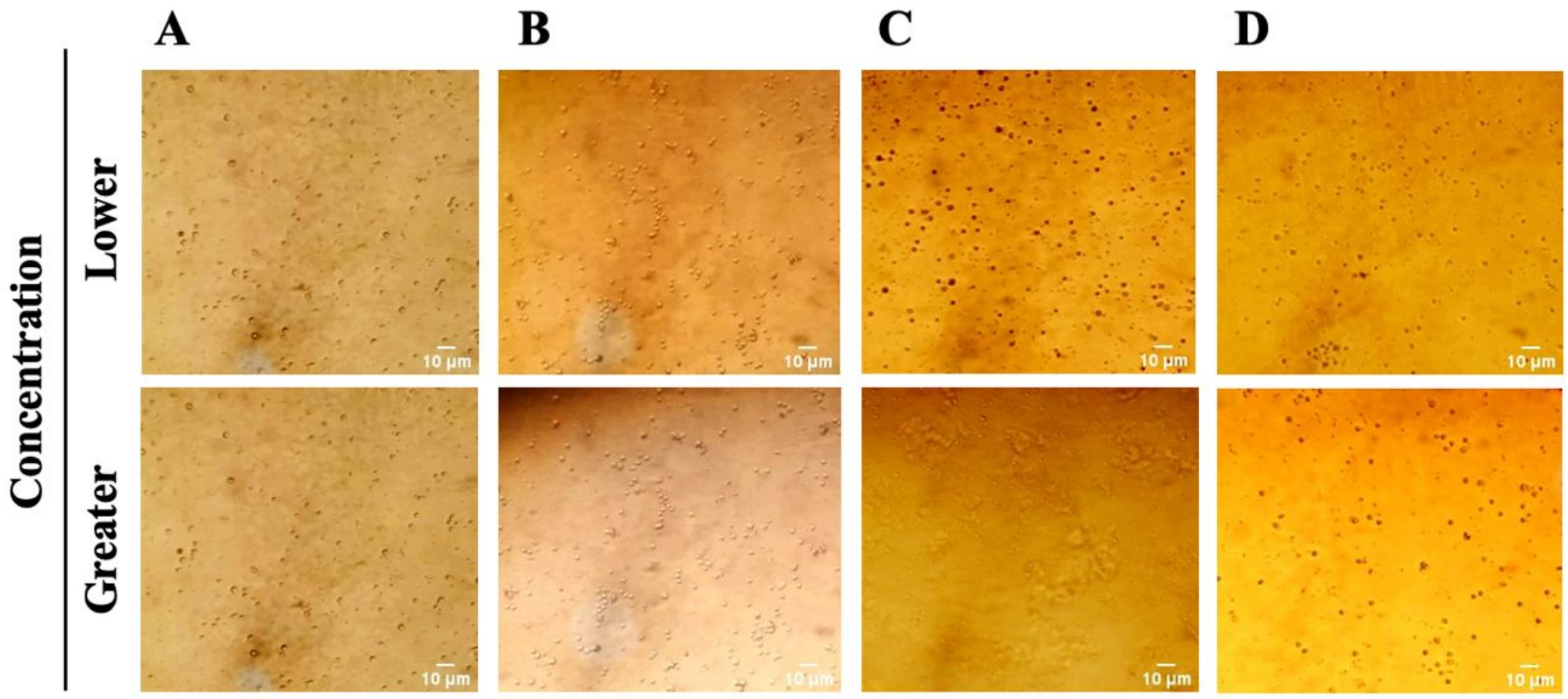

Figure S5. Macroscopic aspects of PBMC cells (A) and after exposure to Lev-NPs (B), Chi-NPs (C), and Fuc-NPs (D) at lower (first row) and greater (second row) concentrations in an optical microscope (10x objective).
